# Advancing Treatment Options for Merkel Cell Carcinoma: A Review of Tumor-Targeted Therapies

**DOI:** 10.3390/ijms252011055

**Published:** 2024-10-15

**Authors:** Helena M. Nammour, Karla Madrigal, Caroline T. Starling, Hung Q. Doan

**Affiliations:** 1 UTHealth McGovern Medical School, Houston, TX 77030, USA; helena.m.nammour@uth.tmc.edu (H.M.N.); karla.madrigal@uth.tmc.edu (K.M.); 2 Department of Dermatology, UTHealth McGovern Medical School, Houston, TX 77030, USA; caroline.t.starling@uth.tmc.edu; 3 Department of Dermatology, Division of Internal Medicine, The University of Texas MD Anderson Cancer Center, Houston, TX 77030, USA

**Keywords:** Merkel cell carcinoma, targeted therapies, receptor tyrosine kinase inhibitors, somatostatin analogs, clinical trials

## Abstract

Although rare, Merkel cell carcinoma (MCC) is a highly aggressive and increasingly prevalent neuroendocrine cancer of the skin. While current interventions, including surgical resection, radiation, and immunotherapy have been employed in treating many patients, those who remain unresponsive to treatment are met with sparse alternatives and a grim prognosis. For this reason, it is of interest to expand the repertoire of available therapies for MCC patients who remain resistant to current primary interventions. Recently, our improved mechanistic understanding of aberrant cell signaling observed in both MCPyV-positive and -negative MCC has facilitated exploration into several small molecules and inhibitors, among them receptor tyrosine kinase inhibitors (TKIs) and somatostatin analogs (SSAs), both of which have positively improved response rates and reduced tumor volumes upon application to treatment of MCC. The introduction of such targeted therapies into treatment protocols holds promise for more personalized care tailored towards patients of diverse subtypes, thereby improving outcomes and mitigating tumor burden, especially for treatment-resistant individuals. In this review, we characterize recent findings surrounding targeted treatments that have been applied to MCC and provide an overview of emerging perspectives on translatable options that can be further developed to offer additional therapeutic avenues for patients with the disease.

## 1. Introduction

Merkel cell carcinoma (MCC) is a rare but aggressive neuroendocrine skin cancer that poses significant treatment challenges due to its propensity for recurrence and metastasis [1]. Originally described as a tumor of epidermal Merkel cells, research on MCC has revealed that pluripotent dermal stem cells may be involved in the pathogenesis of the disease as well [1]. MCC may be triggered by the presence of Merkel cell polyomavirus (MCPyV) in combination with other types of cellular dysfunction, such as damage introduced through ultraviolet (UV) ray exposure or immunosuppression [1]. The incidence of MCC is increasing, attributed partly to an aging population and increased UV exposure, making it a concern of growing importance within the fields of dermatology and oncology [2].

Historically, the mainstay of treatment for MCC has included surgery, radiation therapy, and chemotherapy [1,3]. However, these approaches often yield limited success in the setting of metastatic disease, highlighting the need for novel therapeutic approaches [4,5]. Great progress has been made with the introduction of immunotherapy in treating MCC [3,6,7]. Many patients show durable responses when treated with PD-1 inhibitors, including pembrolizumab, nivolumab, or avelumab. Still, nearly 50% of patients with MCC do not respond to PD-1 checkpoint inhibition [6,7,8]. Furthermore, alternative treatments are needed in patients who may have contraindications to immune checkpoint inhibitors, such as those with severe autoimmune disease or concurrent lymphoproliferative disorders [7]. Recent advancements in the understanding of the molecular pathogenesis of MCC have led to the exploration into and emergence of targeted therapies, marking a new era in the approach to MCC treatment [9]. These therapies are specifically designed to target molecular pathways or genetic mutations that play a crucial role in MCC, offering a more targeted approach [2].

The discovery of MCPyV in the majority of MCC cases and the identification of key molecular pathways involved in the disease have been instrumental in the continuing development of molecularly targeted therapies [10]. Several studies have demonstrated the relevance of the PI3K/AKT/mTOR pathway and angiogenesis-related pathways involving VEGF and PDGF receptors in MCC [11,12]. Differential mechanisms of activation in these pathways among MCPyV-positive and MCPyV-negative MCCs have also been observed. This suggests distinct oncogenic mechanisms in MCPyV-positive versus MCPyV-negative MCCs. The differential pathway activation in MCPyV-associated and non-MCPyV-associated MCCs underscores the possibility of tailored treatments based on the MCC subtype [13]. Ongoing research into molecularly targeted agents, such as tyrosine kinase inhibitors (TKIs) and antiangiogenic drugs, holds promise for expanding the therapeutic options available for MCC. Continued research and clinical trials are essential for fully understanding the potential of these targeted therapies and to integrate them effectively into clinical practice. In this systematic review, we provide a comprehensive overview of the current state of knowledge of the available targeted therapies for MCC and future directions in its management.

## 2. Materials and Methods

We conducted an advanced PubMed search for the following terms, yielding 353 studies conducted between 2004 and January 2024: “Merkel cell carcinoma”, “targeted therapy”, “targeted treatment”, “pazopanib”, “cabozantinib”, “imatinib”, “idelalisib”, “axitinib”, “lenvatinib”, “everolimus” “sunitinib”, “lanreotide”, and “IMGN901”. Publications that included the terms “Merkel cell carcinoma” and “targeted therapy” and made reference to a specific targeted therapy, such as a few of the ones mentioned in the original search criteria, were included. Excluded articles were those that focused solely on immunotherapy, could not be translated into English, or failed to mention a specific targeted agent in the setting of MCC. The final search was narrowed down to 116 articles.

## 3. Overview of the Different Targeted Therapies

In this review, we analyzed 116 articles that discuss the use of various small-molecule inhibitors in the setting of MCC treatment. These agents specifically play a role in targeting cellular pathways that have been aberrantly upregulated or mutated as a result of MCC (Table 1). While 21/28 clinical trials and case reports identified here investigated the efficacy of TKIs and SSAs in reducing tumor volume for patients with MCC (Table S1), laboratory studies also revealed antiapoptotic inhibitors, epigenetic modifiers, p53 activators, PI3K inhibitors, and many other agents to be effective at promoting apoptosis in MCC cell lines as well. The potential for the application of these small-molecule inhibitors in MCC therapy is discussed in the following sections.

## 4. Receptor Tyrosine Kinases

Receptor tyrosine kinases—including VEGFR, PDGFRA, and KIT (CD117)—normally function in the regulation of cell growth and proliferation by translating extracellular signals into activated intracellular messages [45]. These intracellular messages then amplify secondary messengers that ultimately promote expression of growth and proliferation genes, such as vascular endothelial growth factor (VEGF), an angiogenic factor, as well as stem cell factor (SCF) and PDGFA [45]. The dysregulation of the MAP kinase pathway or mutations of the tyrosine kinase receptors can promote a continuous unregulated growth of cells and subsequent tumorigenesis [46]. For this reason, receptor tyrosine kinase inhibitors (TKIs) have been employed in the treatment of various solid cancers, including in the setting of MCC. Specifically, agents such as imatinib, pazopanib, apatinib, lenvatinib, and cabozantinib are TKIs that function as anti-proliferative and antiangiogenic factors to both suppress the metastatic potential of cancer cells and turn off constitutive tyrosine kinase activity (Table 1). The results of preclinical and clinical studies that demonstrate the efficacy of these agents in the treatment of MCC are summarized below (Table S1).

### 4.1. Imatinib

Imatinib is a highly specific small-molecule inhibitor that targets receptor tyrosine kinases such as c-KIT (CD117), BCR-ABL, and PDGFRA [14,47]. This agent has been traditionally applied in the treatment of chronic myeloid leukemia (CML) (Table 1) [48]. While the application of imatinib has shown success in treating some cases of MCC, its role as a therapy for MCC remains controversial, as its efficacy remains equivocal [48].

#### 4.1.1. Preclinical Mechanistic Studies on Imatinib Therapy in MCC

The use of imatinib to treat MCC has been traced back to its selective inhibition of the tyrosine kinase receptor, c-KIT. Feinmesser et al. identified expression of c-KIT in 84% of primary MCC cases and highlighted c-KIT as a potential target for MCC treatments [49].

In MCC tumor cell lines, KIT was found to be activated via an autocrine mechanism through the co-expression of KIT and its ligand SCF. More specifically, the SCF ligand modulates KIT expression by activating downstream signaling pathways critical for cell proliferation [50]. This downstream activation of KIT highlights the potential self-stimulating growth mechanism in MCC cells, independent of external growth signals [50]. As a result of this upregulated pathway leading to cellular proliferation, targeting of the KIT receptor may hold promise as an effective treatment method for patients with MCC.

KIT tyrosine kinases, critical in the oncogenesis of imatinib mesylate-sensitive gastrointestinal stromal tumors (GISTs), suggest the possibility of using imatinib in MCC therapies as well. This approach is based on the premise that similar mutations in MCC, like those in GISTs, could render the cancer cells susceptible to existing kinase inhibition [51].

Further mechanistic studies involving GISTs revealed that 88.2% of GISTs contain active mutations in the KIT gene, notably mutations found in exons 9 and 11. Of note, exon 11 demonstrated a higher rate of partial response (83.5%) to imatinib treatment. In contrast, treatment of tumors with an exon 9 mutation resulted in a lower response rate (47.8%) and no response in cases where no mutation was detected [52].

The presence of common mutations in exons 9 and 11, which have been associated with response to TKIs, has not been observed in c-KIT-positive MCC samples. In one study performed on c-KIT expression and mutations in nine primary MCC tumors, no mutations were found in the examined exons (9, 11, 13, and 17). This absence of mutations raises questions about the role of the KIT receptor in MCC pathogenesis and the potential efficacy of targeted therapies like imatinib mesylate [53].

These findings suggest that while c-KIT is a potential target, its use as a biomarker to underscore the efficacy of imatinib might be limited without the presence of these specific mutations [54]. The lack of a correlation between KIT expression and clinical outcomes, coupled with the lack of activating KIT mutations in MCC, suggests that KIT signaling might not be a significant pathway in MCC tumorigenesis [45,55].

#### 4.1.2. Clinical Trials and Case Studies Reporting the Efficacy of Imatinib Therapy in MCC

The role of c-KIT upregulation in MCC cell lines led to a 2010 phase II trial examining the clinical efficacy of imatinib in patients with metastatic or unresectable MCC. The study enrolled patients who had biopsy-proven metastatic or surgically unresectable MCC who were treated with imatinib mesylate at a dose of 400 mg per day. The results, however, indicated that imatinib mesylate had minimal activity against MCC, despite the expression of CD117 (c-KIT). The study found no complete responses and only one partial response in the 23 study patients, with a 4% objective response rate [14].

Although this phase II trial resulted in minimal response to imatinib treatments, in a case study investigating the use of imatinib in the treatment of MCC, a 77-year-old man with MCC was treated with 400 mg of imatinib twice daily for 6 months, and this dose was subsequently reduced to 400 mg once daily due to clinical intolerance evidenced by grade 3 ageusia and asthenia. The patient underwent surgical excision at three months due to a mixed response characterized by an increase in the size of the main nodule, stability of a frontal nodule, and disappearance of all other lesions. Despite an initial localized relapse at six months, which was treatable with radiotherapy, the continuation of imatinib therapy for an additional three months led to a favorable outcome. The patient experienced no recurrence of disease for 16 months following the cessation of imatinib treatment [56].

In another case study, a 92-year-old female with MCC received KIT-positive tumor-initiated therapy with imatinib 400 mg daily. After two months of imatinib therapy, clinical and radiological evaluations showed complete remission of both the primary tumor and associated metastatic ipsilateral cervical lymph nodes. Despite imatinib’s typically low efficacy in MCC, this case demonstrates an anomalous, robust response, underscoring the potential for imatinib in select MCC patient profiles [48].

In summary, while mutations within the c-KIT receptor have been established as a link for understanding the efficacy of imatinib therapy in MCC patients, the high variability in responses to the agent indicates the further need to identify biomarkers that can more accurately predict whether specific MCC patients will respond better than others to imatinib. As such, the use of imatinib to treat MCC may offer an additional therapeutic option for treatment-resistant patients due to its prior success in treating patients in the few reported cases here; however, providers should be mindful of the less favorable outcomes as described in the phase II trial [14].

### 4.2. Pazopanib

The expression of VEGF-A, VEGF-C, VEGF-R2, PDGF-a, and PDGF-B points towards the involvement of angiogenic pathways in MCC. These proteins could be potential targets for antiangiogenic therapies or TKIs that target these pathways [54]. Pazopanib targets several of these pathway receptors, including the vascular endothelial growth factor receptor (VEGFR) and platelet-derived growth factor receptor (PDGFR), and has been thought to show promise in preclinical studies due to its potent antitumor and antiangiogenic activity [57,58]. Our literature search, however, revealed a handful of case reports with positive results although also some larger studies where patient outcomes were less favorable.

The use of pazopanib in the treatment of MCC was demonstrated in a 69-year-old woman with recurrent, metastatic MCC. After starting pazopanib, CT scans showed a complete response in the patient’s scalp lesion and a partial response in pulmonary metastases, indicating a reduction in the size of the target lesions in the lungs by 57%. The patient experienced minimal adverse effects from pazopanib and maintained a partial response for six months, despite requiring dose reduction [59].

Another investigation of the efficacy of pazopanib in treating four MCC patients with advanced stages of the disease demonstrated highly varied responses upon treatment with pazopanib [60]. Patients receiving pazopanib treatment presented with advanced, metastatic MCC. An initial response was observed for all four patients, as determined by regression of metastatic lesions through FDG PET-CT imaging. One patient, a 61-year-old man, even experienced progression-free survival for seven months while on pazopanib. Eventually, however, all four patients receiving pazopanib in combination with octreotide experienced resistance to the combination along with disease progression [60].

In addition to these case reports, pazopanib has also been investigated in two clinical reports. A phase II trial studying pazopanib in patients with advanced MCC who had been either pre-treated with chemotherapy or were treatment-naive was prematurely stopped. Of the 16 patients that participated, 3 exhibited partial response and 6 experienced disease stabilization. As such, clinical benefit was described for a total of 9 out of 16 patients (56%) [5,61]. The treatment was discontinued in the majority of patients, however, due to progression or toxicity, as well as one death due to gastrointestinal bleeding [61].

In a single-institution retrospective chart review, 18 patients with advanced and pre-treated MCC were identified who were previously treated for a median duration of 8 weeks with different receptor TKIs and targeted therapies either alone or in combination. The TKIs tested included pazopanib, everolimus, lenvatinib, sunitinib, and imatinib. A total of 4 out of 11 (36%) patients who achieved objective stable disease progression for 7–13.6 months following therapy were treated with pazopanib [62]. Genomic profiling was conducted on 3 out of 21 patients who had responded positively to pazopanib, with the aim of identifying any correlations in their mutational profiles. No correlation, however, was found in the mutational landscape of the three tumors included in this small retrospective analysis investigating 332 cancer-related genes. This lack of correspondence between the three patients receiving next-generation sequencing (NGS) suggests a further need to understand what factors drive a response to pazopanib therapy in MCC patients. One finding suggested that patients who received prior treatment with immune checkpoint inhibitors were more likely to exhibit similar genetic profiles and a more beneficial response to pazopanib treatment [62].

Overall, among the two case reports and two clinical studies identified here, a variety of responses were observed for MCC patients receiving pazopanib treatment. While some patients experienced stable disease progression, others did not benefit from the intervention, and one clinical trial was prematurely discontinued for 15 out of the 17 evaluable patients due to an imbalance between toxicity and the positive response achieved from pazopanib therapy [61].

In both clinical reports, the median duration of therapy was 8 weeks. Additionally, all individuals receiving therapy were advanced-stage MCC patients, and response definitions were derived from the Response Evaluation Criteria in Solid Tumors (RECIST) guidelines version 1.1. The main difference found among both studies pertain to the time points at which clinical responses to pazopanib were evaluated. In the phase II trial, responses to treatment (either partial or complete) were reported at 12 weeks following therapy, whereas in the retrospective review, responses were determined following 6 months of pazopanib treatments [61,62]. The different methodological approaches used to evaluate partial and complete responses may affect how these results were portrayed.

Although prior research has revealed the inefficacy of pazopanib in treating MCC patients with advanced disease, it remains unclear whether targeting angiogenic pathways in MCC may still mitigate the extent of metastasis in less advanced cases. As such, more drug combinations including pazopanib should be explored to understand how they may synergize and provide an effective response in MCC patients.

### 4.3. Cabozantinib

Cabozantinib is an oral, small-molecule inhibitor targeting multiple tyrosine kinases including c-MET and VEGFR-2 (Table 1). There have been mixed outcomes in patients with MCC who were treated with cabozantinib in published case reports, case series, and one phase II trial.

In a case series involving the treatment of neuroendocrine tumors with TKIs, a 69-year-old man achieved prolonged disease control of about 3.5 years upon receiving cabozantinib treatments. After recurrence, he switched to nivolumab and radiation therapy [60]. In another case, an 86-year-old man with metastatic medullary thyroid cancer was diagnosed with another primary tumor, a stage III MCC of the right leg. He underwent resection of the primary MCC followed by adjuvant locoregional therapy [16]. Treatment with 100 mg cabozantinib daily resulted in side effects, such as weight loss, fatigue, dysgeusia, mild hypocalcemia, elevated blood pressure, and moderate lymphopenia. A reduction in the daily dose led to a partial response complicated by leukopenia; however, years after his initial MCC diagnosis, he showed a complete response according to RECIST version 1.1 criteria [16].

In a prospective phase II trial evaluating the efficacy and safety of cabozantinib in patients with advanced MCC who previously failed platinum-based therapy, six males and two females, with a median age of 66.5, received a daily oral dose of 60 mg of cabozantinib until disease progression or the development of significant toxicity. The most positive response observed was stable disease progression for 8 months in one patient, as overall cabozantinib showed limited activity and was poorly tolerated [63].

The presence of adverse effects and lack of robust, positive results from clinical trials suggest that more research must be conducted before introducing cabozantinib as a viable and comparable alternative to other MCC treatment options. Rather, cabozantinib may hold more promise in treating solid tumors such as medullary thyroid cancer, hepatocellular carcinoma, and renal cell carcinoma [46].

Beyond imatinib, cabozantinib, and pazopanib, researchers have also investigated the efficacy of different TKIs, such as apatinib, an antiangiogenic VEGF-2 inhibitor, in MCC treatments (Table 1). Specifically, 5 months of treatment with 250 mg apatinib and endostar resulted in progression-free survival for 6.5 months in an 86-year-old patient with MCC [17,64]. Although a partial response was observed, combinations of TKIs and other targeted therapies should nevertheless be explored to offer maximum therapeutic benefit to patients with the disease.

In addition to apatinib, there are two ongoing trials that will explore TKIs as multimodal therapies, including one with axitinib (VEGFR-1,2,3 inhibitor) with sansanlimab (anti-PD1 antibody) and PF-07265807, which targets AXL and MERTK, thereby inhibiting tumor-associated macrophage kinases (NCT04458259; active, not recruiting), as well as another phase II trial exploring pembrolizumab with lenvatinib (VEGFR, FGFR, and RET inhibitor) given 6 weeks prior to surgery in patients with MCC (NCT04869137) [5]. In summary, given the mixed outcomes in monotherapy with TKIs in MCC and several phase II trials with progression or significant toxicities, studies focusing on TKI as multimodal therapies are warranted.

## 5. Somatostatin Analogs

Another group of drugs previously investigated in the treatment of MCC are somatostatin analogs (SSAs). SSAs bind specifically to somatostatin receptors (SSTRs) to prevent tumor-activated endocrine regulators that result in tumor growth, such as insulin, gut hormones, TSH, and growth hormones (GHs) (Table 1) [19,65]. It has been reported that ~90% of neuroendocrine tumors (NETs) carry expressions of SSTRs [66]. In a study that sequenced 39 MCC tumors, SSTRs were identified in ~85% (33/39) of the samples. As a result, the use of SSAs has been expanded in clinical treatments of MCC [67]. Octreotide and lanreotide are two SSAs that have been employed in clinical trials to treat patients with MCC.

Even before researchers understood the comprehensive mechanism by which SSAs operate to eliminate MCC tumor cells, these targeted agents have been applied to therapies. In 1997, a patient with metastatic MCC showed a complete response to octreotide treatment lasting ten months, thus suggesting the therapeutic potential of SSAs in the setting of MCC [68,69].

More recently, a 2020 report described a 73-year-old man with recurrent MCC who was treated with octreotide every 28 days for 2 years after he had experienced disappointing results with radiation and chemotherapy during the treatment of his primary tumor [65]. The patient’s disease progressed while on octreotide monotherapy; however, when avelumab was added to the regimen, a complete remission of the tumor was observed, as determined by a CT scan 17 months following the introduction of the combination. Despite achieving complete regression of the metastasized MCC lesion, the patient continued receiving the octreotide and avelumab combination without remarkable side effects. This report introduces the possibility of combining SSAs with certain immunotherapies, as these agents may promote synergistic effects and enhance the immune response to MCC [65].

A retrospective study that explored the efficacy of octreotide in treating patients with metastatic MCC and found that, out of 19 patients treated with octreotide, 7 responded positively, with 3 patients exhibiting progression-free survival over an average of 237 days [67,70].

In the case of an 87-year-old female patient with disseminated, inoperable MCC, treatment with lanreotide, another SSA, led to complete remission over a period of 17 months. The patient responded remarkably and without side effects, despite experiencing recurrence of the disease after three separate surgical excisions [68,71].

Not only have SSAs been investigated therapeutically as monotherapies, but they also have been cross-linked to radiolabels to provide more selective diagnostic tools to visualize metastasis in patients with MCC [66,68,72,73]. In one case of an 81-year-old female with recurrent and refractory MCC, yttrium-90-radiolabeled somatostatin analog (^90^Y-DOTATOC) was selected due to SSTR expression on MCC and the patient’s ineligibility for conventional chemotherapy as a result of age and associated risks. The patient experienced complete clinical remission within a week after the initial dose, and subsequent treatments led to periods of remission, demonstrating the potential efficacy of ^90^Y-DOTATOC as a targeted therapy for MCC [66].

Finally, peptide receptor radionuclide therapy (PPRT) is a treatment that uses a radiolabeled SSA that is metabolized by the tumor to produce local radiation that kills surrounding tumor cells [5,74]. These agents include 177Lu-DOTATATE and ^68^Ga-DOTA-Tyr3-octreotide. A phase II trial was performed to assess efficacy and toxicity in many types of neuroendocrine tumors, and although its results are not available, this type of therapy should be investigated as a possible alternative to treatment for MCC [75]. There is a clinical trial (NCT04261855) examining combination therapy with avelumab and 177-Lu-DOTATATE or external beam radiation currently in progress.

Overall, we identified a few case studies reporting the effects of SSA therapy on MCC patients. Such reports revealed that SSAs are capable of promoting stabilization of disease progression in patients with the disease. Although such case reports carried a positive impression of SSAs as a potential therapy for treatment-resistant patients, there still remains a relative lack of information explaining the range of responses experienced by patients taking SSAs, as well as robust clinical trials that provide convincing data on the efficacy of such agents. As such, future research should work not only to explain the deficits of SSA therapy in the setting of MCC but also to identify potential biomarkers that may be used to predict which patients would receive more benefit from using SSAs over other interventions. Additionally, more clinical trials must be performed to estimate the efficacy of SSAs in patients with MCC. As such, not only can SSAs play a role in treatment of the disease, but studies show they may be useful as radiolabels to be combined with positron emission tomography (PET) to better mark regions of metastasis in MCC patients. Such use of SSAs may prove more effective than conventional CT scanning techniques [68,72,73].

## 6. Antiviral Agents

Antiviral agents are a group of drugs that specifically target MCPyV-positive cases of MCC, as they work to inhibit the proliferation of the virus in tumor cells as well as subsequent tumorigenesis. Many antiviral agents have been applied to in vitro treatments of MCC in the past; however, clinical evaluations of these therapies remain scarce.

Prior studies have shown that the large and small T antigens (LTA and STA, respectively), mediators of cell growth in MCPyV-positive MCC, play key roles in disrupting cell cycle regulation. Specifically, LTA binds and inactivates the RB1 protein, leading to unregulated progression through the cell cycle, while STA enhances cell proliferation by inhibiting PP2A. For this reason, inhibiting LTA and STA function is of interest, as many established MCC cell lines require expressions of these viral proteins for continued proliferation [40,41].

The suppression of LTA and STA activity has been effective in the past via a number of agents. Specifically, trametinib, a specific inhibitor of the mitogen-activated protein kinase enzymes MEK1 and MEK2, has shown promise by indirectly affecting viral protein expression. MEK inhibition disrupts the MAPK/ERK pathway, which is involved in cell proliferation and survival [42]. By inhibiting this pathway, trametinib reduces the expression of MCPyV LTA and STA, crucial for viral-driven oncogenesis in MCC [42]. Liu et al. demonstrated this effect in cell line studies, showing that MEK inhibition can suppress viral load in patients who are immunocompromised [42,76]. In addition, Selinexor, an XP01 inhibitor, displayed antiviral activity against MCPyV in vitro via dose-dependent downregulation of both LTA and STA [41].

More recently, it has been found that the glycogen synthase kinase 3 (GSK3) operates in the STA regulation pathway and can be inhibited in order to suppress STA expression. Inhibition of GSK3 with CHIR99021 in vitro and in vivo xenotransplanted mice revealed repressed growth of tumors compared with the vehicle, although tumor growth did not stop altogether. Importantly, daily treatment in mice with CHIR99021 did not precipitate any visible toxic side effects, indicating its potential to be translated into clinical settings [76]. In addition to GSK3 inhibition, it was found that the inhibition of p300 and CBP histone acetyltransferase (HAT) with various HAT inhibitors halts MCPyV tumor antigen expression and virus-positive MCC growth in cell line experiments [77].

Antiviral therapies, although functional only in MCPyV-positive MCC cases, remain fascinating to explore as potential targeted therapies for MCC upon more thorough investigation into clinically translatable forms of these therapies.

## 7. PI3K/mTOR/AKT Pathway Agents

PIK3CA encodes for the p110α subunit of PI3K, a key component of the PI3K/pAKT pathway. This pathway is instrumental in cell growth and survival and is often dysregulated in cancers. In MCC, the presence of these mutations, particularly in the helical and kinase domains, suggests an oncogenic role for the PI3K/pAKT pathway [78]. Previous studies have shown that the PI3K/mTOR/AKT pathway is aberrantly upregulated in MCC. As such, the constitutively active proteins within this pathway may serve as potential targets for treatment of the disease [33,79].

### 7.1. Preclinical Studies Focusing on the PI3K/mTOR/AKT Pathway

In vitro and in vivo treatment of MCC mouse models with a panel of PI3K inhibitors revealed markedly reduced tumor size and upregulated expression of caspase-3, indicating apoptosis activation in MCC cells when copanlisib was applied [33,79]. Other preclinical studies on mTOR inhibitors have shown promising results, including specific mTOR inhibitors such as WYE-354, Ku-0063794, and PP242 in MCC cell lines. These inhibitors effectively suppressed mTOR pathway activation, evident from the reduced phosphorylation of key proteins. The study also demonstrated an induction of autophagy, or lysosomal-dependent degradation of intracellular contents, as well as cell death in MCC cells treated with these inhibitors, a process independent of caspase activation [80].

Several other inhibitors examined in preclinical studies have shown successful repression of MCC cells. This includes combination therapy with alpeselib (PI3K-alpha inhibitor) and navitoclax (BCL-2 inhibitor), resulting in a synergistic effect that decreased MCC cell survival [34]. Afuresitib is an AKT inhibitor that results in decreased MCC cell growth as well [81]. Similarly, Koubek et al. showed that synthetic schweinfurthin represses MCC cells via the AKT signaling pathway [82].

In vivo treatments with MLN0128, an mTOR inhibitor that exhibits antiproliferative activity, were successful in attenuating the growth of tumors and inducing apoptosis in mouse models injected with three different MCPyV-positive MCC cell lines [35]. MLN0128 has also been shown to have a synergistic treatment effect when combined with trametinib (MEK 1/2 inhibitor) in another MCC cell line study [83].

### 7.2. Clinical Studies Focusing on the PI3K/mTOR/AKT Pathway

A phase I clinical trial assessed the safety and tolerability of MLN0128 in nine patients with MCC (NCT02514824). In the study, four patients were treated with 3 mg of the drug orally, while five patients were administered 4 mg indefinitely. Of the nine patients included in the trial, one participant in the 4 mg arm eventually withdrew due to toxicity [84]. Due to limited participant accrual and a lack of efficacy, this trial did not proceed to phase II.

Idelalisib is a selective phosphoinositide 3-kinase (PI3K)δ inhibitor that also functions in the PI3K/AKT pathway [85]. In a report on an 86-year-old Caucasian woman with stage IV MCC who had undergone surgery and radiation, a complete clinical response was achieved with idelalisib treatment. One week after initiation, regression was observed in a metastatic liver lesion. This response was followed by no observable tumor upon PET-CT three months later, thus indicating a complete clinical response to the therapy [85,86].

In general, there is a lack of convincing clinical data that highlight the efficacy of PI3K/mTOR/AKT pathway inhibitors in the treatment of MCC. Specifically, mTOR inhibitors such as MLN0128 did not prove tolerable nor effective as a therapy for MCC patients. Regarding PI3K inhibitors, however, we noted that synergistic combinations of drugs such as idelalisib with other agents may promote the obliteration of tumor cells in treatment-resistant patients and should be further investigated in clinical trials [34].

## 8. Epigenetic Modification

Epigenetic modification functions to derepress proteins whose activity have been compromised by tumor cells. Ultimately, re-expressing such proteins can function to eliminate cancer cells as a result. The study of histone post-translational modifications in MCC is a growing area of research with several analyses demonstrating successful MCC cell death when various proteins in this process are inhibited [87]. Previous studies have specifically examined the preclinical efficacy of LSD1, HDAC, EZH2, and BET inhibitors on MCC cell lines and mouse studies to understand whether these agents hold the potential for clinical translation.

Lysine-specific histone demethylase 1a (LSD1) is an epigenetic modifier that removes methylation marks on the H3K4 histone and indirectly promotes the proliferation of MCC tumor cells. Leiendecker et al. investigated the effects of LSD1 inhibition in a mouse xenograft model of MCC and found that treatment with GSK-LSD1 for 6 days decreased tumor size. Additionally, the authors found increased apoptosis in MCC cells when treated with GSK-LSD1 for 24h as compared with 0.9% saline vehicle-treated mice [88].

Another study performed in 2020 similarly demonstrated that the inhibition of LSD1 decreases MCC growth through a CRISPR-Cas9 screen [86,89]. When combined with immune checkpoint inhibitors, LSD1 inhibition has been found to induce interferon 1 signaling and enhance tumor response to therapy, making the agent a potential supplement to current immunotherapies approved for the treatment of MCC [88].

Domatinostat is a histone deacetylase inhibitor (HDACi) that induces the transcription of repressed genes and promotes G2/M phase arrest and apoptosis in MCC cells. Domatinostat has been shown to upregulate antigen presentation on MCC cells in vitro, which can promote the efficacy and prevent resistance of certain immunotherapies [23,87]. The MERKLIN 1 study explored the role of domatinostat in combination with avelumab in MCC; however, the trial was ultimately withdrawn due to a refocusing of the clinical development program, and this withdrawal was unrelated to safety concerns or changes in the risk–benefit assessment of the investigational drug [5]. MERKLIN 2 (NCT04393753P) is an ongoing trial of domatinostat and avelumab for advanced stage III and IV MCC patients who had progressed while receiving prior PD-1 antibody monotherapy. The results for MERKLIN 2 have not yet been published; however, the goal of MERKLIN2 is to understand whether the 19 patients currently enrolled will experience a complete or partial response (for up to 24 months) to the avelumab and domatinostat combination based on RECIST v1.1 criteria.

Other HDACi include Panobinostat, which was shown to have an increase in HLA class I expression and CD8+ T-cell infiltration upon treatment of MCC tumor cells, though it did not seem to have a major clinical impact on two patients that had failed PD-1/PDL-1 blockade [25]. Additionally, treatment of MCC cells in vitro and in vivo with vorinostat (another HDACi) and mithramycin A induced MHC class I protein expression and thus can potentially play a role in modulating the response to immunotherapy [90].

EZH2 is a subunit of polycomp repressive complex 2, which is involved in chromatin compaction and gene silencing. Low EZH2 expression correlates with poor prognosis in patients with MCC while high EZH2 expression indicates metastasis or recurrence [91]. Inhibition of EZH2 would allow for decreased expression of histone methyltransferase, an enzyme responsible for silencing different genes.

EZH2 inhibitors have been studied in other cancers such as epithelioid sarcoma as well as some leukemias and lymphomas. There have been mixed outcomes in MCC cell lines; however, some studies have suggested that EZH2 inhibition may be a negative regulator of virus-positive MCC [5,92]. In a study evaluating the effectiveness of treating MLK-1 xenograft tumors in a mouse xenograft model with Tazemetostat—an EZH2 inhibitor—for 50 days, showed a significant attenuation of tumor growth by day 19 of treatment; however, a complete cessation of tumor growth was not achieved [26]. Continued research on EZH2 inhibition as a treatment for MCC is warranted to understand the mechanistic role of EZH2 in the activity of MCPyV-positive MCC; however, the incomplete elimination of tumor growth in vivo points to the need to study EZH2 inhibition in combination with other therapies to promote synergy and cell death [26].

Finally, the bromodomains and extra-terminal (BET) family of proteins are involved in chromatin acetylation and the induction of genes promoting cell survival and proliferation. Knockdown of the BET protein BRD4 in acute myeloid leukemia through RNA interference has previously led to decreased expressions of c-Myc, a master regulator of proliferation. Due to the high expressional profile of c-myc in MCC, BET proteins have presented as another potential target of therapy to be explored in the case of MCC [32].

In a study investigating the treatment of MCC cell lines with BET protein inhibitor JQ1, a significant reduction in c-Myc expression was observed in MCC cell lines, accompanied by a dose-dependent inhibition of cell proliferation [32]. The treatment also induced cell cycle arrest and decreased colony formation. Importantly, JQ1 significantly impaired the growth of MCC xenograft tumors in vivo as well. These findings establish the therapeutic potential of BET protein inhibitors like JQ1 in the management of MCC, especially in cases with abnormal c-Myc activation [32].

In vitro and in vivo applications of BETd-246, another BET degrader, in the treatment of 16 MCC cell lines revealed reduced tumor volume of cells following treatment for 16 days in a mouse xenograft model [31]. Additionally, BETd-246 showed a reduction in MCC tumor volume comparable to the BET inhibitor OTX-015 even when administered at lower concentrations.

## 9. Proapoptotic Agents

### 9.1. Bcl-2 Inhibitors

Bcl-2, a key protein in the intrinsic apoptosis pathway, is notably overexpressed in about 80% of MCC cases [15]. This overexpression contributes to the low levels of apoptosis characteristic of MCC. ABT-263 is a Bcl-2 family inhibitor that binds to Bcl-2, Bcl-xL, and Bcl-w, disrupting their interaction with proapoptotic proteins and inducing apoptosis in MCC cells [93]. Oblimersen, an antisense oligonucleotide, was successful in preclinical studies but failed in clinical trials [92]. Targeted therapies against BCL-2, like Oblimersen sodium and ABT-263, have shown varying degrees of preclinical effectiveness [15].

Bcl-2 is an antiapoptotic protein that can be upregulated in various cancers to promote tumor survival and prevent cell death, making Bcl-2 another possible target for treatment. Although previous studies mention the inefficacy of Bcl-2 inhibitors when applied as monotherapies, these agents have been successful in sequential and combined treatments as senolytic agents that specifically target senescent cells for death [10]. In vitro treatment of MCC cells with ABT-199—a bcl-2 inhibitor—and glaucarubin revealed markedly reduced viability in five out of the eight MCPyV-positive cell lines tested [10].

Previous studies have attempted the use of BCL-2 antisense oligonucleotides to silence BCL-2 in vivo with mouse models and in a phase II clinical trial; however, these investigations demonstrated very little efficacy in the treatment of MCC [94].

Furthermore, it was found that inhibiting B-cell lymphoma-extra-large (Bcl-xL), which is encoded by Bcl2L1, resulted in antitumor effects in MCC cell lines, including a synergistic effect when poly (ADP-ribose) polymerase 1 (PARP) inhibitors are added [95].

### 9.2. Survivin

Survivin is a member of the inhibitor apoptosis (IAP) family of proteins and functions in regulating mitosis and apoptosis. MCPyV-positive MCC has been found to upregulate survivin transcription through the action of the large T antigen, correlating with a higher expression level in MCPyV-positive MCC compared to MCPyV-negative cases. High survivin levels are associated with poor prognosis and increased chemoresistance in cancers [96]. Targeting survivin could prove to be a promising strategy as it is involved in tumor cell survival.

YM155, a novel survivin transcription inhibitor, has shown promising results in selectively targeting MCPyV-positive MCC cell lines [97]. The mechanism of action of YM155 includes binding to interleukin enhancer binding factor 3 (ILF3) and disrupting transcriptional complexes at the survivin promoter [30]. In vitro, YM155 was effective at low concentrations and caused necroptotic cell death but functions more as a cytostatic agent in vivo, temporarily halting tumor growth without eradicating it. These findings indicate that while YM155 is a potential therapeutic agent for MCC, particularly in virus-positive cases, its efficacy may vary, and further investigation is needed for its application in clinical settings [97]. Molecular inhibitors of survivin, such as YM155, could offer a new therapeutic avenue for patients with strong nuclear survivin expression [98].

### 9.3. Rescuing p53 Function

One study performed in 2013 highlighted the low frequency of p53 mutations in MCC and suggested that p53, a key tumor suppressor and proapoptotic protein, is often functionally suppressed rather than genetically altered in MCC [99]. Their study showed that in most MCC cell lines, p53 is inactivated not by viral proteins from MCPyV but potentially through the action of HDM2, also known as murine double minute 2 (MDM2). HDM2/MDM2 is known to regulate p53 by promoting its degradation. By using Nutlin-3a, a small-molecule inhibitor of HDM2, the authors demonstrate that p53 activity can be restored in MCC cell lines, leading to cell cycle arrest or apoptosis of MCC cell lines [99,100].

Further mechanistic studies identified the role of MDM2 and MDM4 in downregulating p53 expression in MCC cells and found that MDM4, a negative regulator of p53, was upregulated in MCPyV-positive MCC cell lines specifically. The combination of MDM2 inhibitors with lenalidomide or an MDM4 inhibitor in vivo and in vitro rescued p53 function in MCPyV-positive cell lines [27].

Navtemadlin (KRT-232) is an MDM2 inhibitor currently being investigated in a clinical trial (NCT03787602) involving patients with p53 wild-type MCC, both as a monotherapy and in combination with avelumab (PD-L1 inhibitor) [4,27,28]. The dose-finding study presented by Wong et al. at ASCO including 31 patients showed encouraging results with a 25% confirmed objective response rate (ORR) and a 63% disease control rate among those receiving the 180 mg 5 days on/23 days off dose. The most common adverse events were hematologic cytopenias.

## 10. Antibody–Drug Conjugates

Antibody–drug conjugates (ADCs) are monoclonal antibodies (mAbs) covalently attached to cytotoxic agents that are used to target specific intracellular molecules and mediate apoptosis [101]. As of December 2023, there are 14 ADCs currently on the market that treat various cancers, with hundreds more currently under investigation in clinical trials. It is projected that ADCs will eventually replace conventional chemotherapy regimens due to their increased specificity for intracellular targets [102]. Recent studies have shown the efficacy of ADCs in treating MCC by acting on different cellular targets.

One molecule that is consistently expressed in MCC and has been studied as a potential target for ADC therapy is CD56, a neural cell adhesion molecule. Targeting CD56 with ADCs presents a novel therapeutic approach [103]. Lorvotuzumab mertansine (IMGN901) combines an antibody targeting CD56 with a cytotoxic agent, DM1, and has been granted orphan drug status for MCC. The principle behind this approach is to leverage the high expression of CD56 in MCC for targeted delivery of the cytotoxic agent, thereby potentially enhancing the efficacy of the treatment while minimizing side effects [104]. IMGN901 merges a potent microtubule assembly inhibitor derived from maytansinoid with a humanized monoclonal antibody specifically designed to target CD56 [105]. In a phase I study of IMGN901, 4/23 patients demonstrated an objective response based on RECIST 1.0 criteria, and 3 out of 4 who responded to therapy were MCC patients. One MCC patient exhibited a complete response (elimination of all malignant lesions), another demonstrated a complete clinical response, and one partial response with disease progression following two treatment cycles was observed in the third patient who responded positively to IMGN901 [20]. Adverse effects ranged from fatigue and headache to hyponatremia, neuropathy, chest pain, dyspnea, and myalgias.

Adcitmer® is another CD56-targeting antibody and cytotoxic compound (monomethyl auristatin E) that has shown decreased MCC tumor growth in cell culture and mouse models [21]. Notably, Adcitmer^®^ was tested both in vitro and in vivo in an MCC xenograft mouse model to determine the efficacy and toxicity of the drug on treatment of CD56-positive Merkel cell lines. Tumor volume was found to be significantly reduced in mice that were treated with three injected doses of Adcitmer^®^ vs. the vehicle. Additionally, significant weight loss or toxicity were not observed in the mice, indicating the potential for this drug to be used as a nontoxic alternative to treatments for patients with MCC [22].

Beyond CD56 ADC targeting, ABCB5 blockage with an anti-ABCB5 mAb has also shown efficacy in reducing MCC drug resistance to carboplatin and etoposide in vivo, attenuated xenograft growth, and increased caspase 3 expression in mice injected with MKL-1 tumor cells [37].

ADCs are not only currently under investigation as monotherapies, but they are also being applied as combination therapies with immunotherapeutic agents to understand the synergistic effects of the drug regimens. IL-7 increases the number of T-cells and enhances these cells, aiding in antitumor response (Table 1). NT-I7, a more stable, long-acting human IL-7 (efineptakin alfa), for example, is being studied in combination with atezolizumab (NCT03901573) to treat patients with inoperable stage III-IV MCC [4,21].

Taken together, these findings suggest the importance of introducing tolerable agents such as Adcitmer ® into more advanced clinical trials to assess their efficacy in MCC treatments. Due to their selectivity, researchers have found interest in the development of hundreds of ADCs that carry the potential to reduce toxic side effects of generalized chemotherapeutic interventions. While CD56 and IL-7 are two targets of ADCs that have been recently studied in the treatment of MCC, more targets for treatment should eventually be explored as well, especially as the repertoire of available ADC therapies continues to expand over time.

## 11. Other Small-Molecule Inhibitors and Potential Drug Targets

Other small-molecule inhibitors that have been investigated through in vitro studies of MCC cell lines include palbociclib, a CDK 4/6 inhibitor that, when combined with TC-S7009, disrupts hypoxia-inducible factor (HIF)-2 alpha and results in increased reactive oxygen species and ferroptosis (Table 2) [38]. Additionally, Aurora Kinases (AURKs) are enzymes that promote cell survival when activated and indicate poor prognosis when upregulated in different cancer types; consequently, AURK inhibitors may be a reasonable approach to MCC tumor inhibition [39]. In a study of six MCC cell lines treated with AK-01, an AURK inhibitor, cell proliferation decreased by ~50% when MCC cells were treated with concentrations >100 nM. Regarding xenograft models, AK-01 treatment induced progression of the cell cycle to the G2 phase in three MCC cell lines [37].

Here, we described ongoing in vitro, in vivo, and clinical studies that hold the potential for providing MCC patients with additional, effective targeted therapy options. In addition to the previously mentioned targeted therapy trials, current studies have also focused on improving our mechanistic understanding of aberrant cellular pathways in both MCPyV-positive and MCPyV-negative MCCs. These investigations have revealed a number of proteins and pathways that are specifically upregulated or mutated in MCC cell lines (Table 3). Future research should work to understand if targeting these mechanisms leads to significant levels of apoptosis or tumor volume shrinkage in laboratory and ultimately translatable studies including MCC cell lines.

In addition to providing novel perspectives on upregulated and aberrant pathways, many of these studies suggest the use of agents that are currently employed in clinical practice in the treatment of other cancers, such as tesirine, a DLL3 inhibitor used in small-cell lung carcinoma [21], or Poly-ADP inhibitors to target BRCA1/2 mutations in ovarian cancers [106]. The application of previously developed inhibitors could be more easily translatable to clinical trials for understanding the efficacy of such treatments in the setting of MCC, as many of these proposed small molecules have previously established toxicity levels in prior phase I clinical trials [128].

## 12. Conclusions

Merkel cell carcinoma (MCC) is an aggressive neuroendocrine skin cancer that has historically been treated with surgery, radiation therapy, and chemotherapy. While many MCC patients have benefitted from these treatments, those who remain unresponsive are left with limited therapeutic options. For this reason, it is important to continue exploring novel therapies to offer relief to patients who suffer from advanced and resistant forms of the disease.

Unlike traditional chemotherapy, targeted therapies attack specific cellular mechanisms that have been altered during tumor formation and metastasis. The development of targeted agents has, over time, mirrored our improved understanding of the dysregulated mechanisms that impede proper cellular homeostasis and promote oncogenesis. Treatment that is focused on rectifying these oncogenic signaling pathways may provide patients a better prognosis and improved therapeutic response.

The current review has focused on the promise of applying novel targeted approaches in the management of MCC. Specifically, our findings underscore the potential of applying receptor tyrosine kinase inhibitors (TKIs) and somatostatin analogs (SSAs) to the setting of MCC, as these agents have shown improved objective response rates and stable disease progression in cases of advanced MCC. Additionally, we found that treatment of MCC cell lines with various small-molecule inhibitors, such as Bcl-2 inhibitors, epigenetic modifiers, BET degraders, and ADCs, has facilitated apoptosis and tumor volume reduction in MCC mouse xenograft models. Future research must focus on maximizing the efficacy of treatment while minimizing toxicity, as the intolerability of certain treatment regimens was found to be a limiting factor for many of the trials we identified. As such, the application of targeted therapies in the management of MCC remains an exciting and rapidly expanding field of research, and the translation of such targeted therapies into MCC treatment protocols holds promise for more personalized care tailored towards patients of diverse subtypes, thereby improving outcomes and mitigating tumor burden, especially for those who are resistant to conventional interventions.

## Figures and Tables

**Table 1:** General mechanism of action for targeted therapies that have either undergone a series of clinical trials for the treatment of MCC or have previously been tested on Merkel cell carcinoma cells in vitro.

Therapy	Type	General Mechanism	Reference
Imatinib	Receptor TK inhibitor	A small-molecule inhibitor that binds to receptor tyrosine kinases, including those with the c-KIT receptor, CD117.	Samlowski et al. (2010) [14]
Pazopanib		Targets multiple receptor tyrosine kinases, including c-KIT, FGFR, PDGFR, and VEGFR to prevent angiogenesis.	Tothill et al. (2015) [15]
Cabozantinib		Inhibits multiple receptor tyrosine kinases, (VEGFR)-2, tyrosine-protein kinase receptor UFO (AXL), mesenchymal epidermal transcription factor (MET), and rearranged during transfection (RET).	Zago et al. (2024) [16]
Apatinib		VEGFR-2 tyrosine kinase inhibitor that prevents angiogenesis.	Feng et al. (2021) [17]
Cetuximab		Monoclonal antibody that binds and inhibits EGFR, thus preventing proliferation.	Scarpati et al. (2016) [18]
Lenvatinib		Inhibits VEGFR, FGFR, and RET and subsequently halts angiogenesis.	Celikdemir et al. (2023) [5]
Axitinib		Small-molecule inhibitor of VEGFR1, 2, and 3.	Celikdemir et al. (2023) [5]
Lanreotide	Somatostatin analogs (SSAs)	SSAs bind to somatostatin receptors (SSTRs), a family of GPCR, and prevent the release of insulin, gut hormones, TSH, and GH in tumors that promote the synthesis and release of such hormones.	Grimberg et al. (2004) [19]
Octreotide			
90Y-DOTATOC and PPRT			
IMGN901	Anti-CD56 antibody	Used for treatment of CD56-positive cancers. CD56 (NCAM1) is a neural cell adhesion molecule that functions in normal development and is associated with several hematological malignancies and solid tumors. IMGN901 releases DM1 upon binding to CD56 and entering the cell. DM1 disrupts microtubule assembly and induces G2/M phase arrest.	Shah et al. (2016) [20]
Adcitmer®		Used for the treatment of CD56-positive cancers. Adcitmer is an antibody–drug conjugate that combines a CD56-specific mAb with MMAE, an antimitotic drug, to kill cancer cells.	Esnault et al. (2022) [21,22]
Domatinostat	Epigenetic modifier	Domatinostat, a class I HDACi, inhibits the transcriptional silencing of certain genes, whose expression would have been otherwise silenced by a tumor cell. In this way, Domatinostat rescues tumor suppressor gene activity in tumor cells, promotes cell cycle arrest, and induces apoptosis in tumor cells through persistent G2M arrest.	Song et al. (2021) [23]
LSD1		LSD1 removes mono- and demethylation marks on histone H3 lysine 4 (H3K4), which are linked to active transcription. Thus, LSD1 represses gene expression.	Mauri and Blanpain (2018) [24]
Panobinostat		Panobinostat is an HDACi that induces strong expression of HLA molecules in the cell.	Ugurel et al. (2019) [25]
Tazemetostat		EZH2 is a subunit of polycomp repressive complex 2, which is involved in chromatin compaction and gene silencing via histone methyltransferase.	Gartin et al. (2022) [26]
Nutlin-3a	MDM2 inhibitor	Small-molecule inhibitors that bind to the p53 active site in the MDM2 protein and prevents MDM2-mediated targeting of p53 degradation.	Park et al. (2019) [27]
Navtemadlin (KRT-232)			
Lenalidomide	MDM4 inhibitor	Inhibits MDM4 via degradation of Ck1 [28]. Degradation of Ck1 prevents MDM4 interaction with p53 and rescues p53 function in the cell. Lenalidomide has previously been applied in the treatment of multiple myeloma.	Park et al. (2019), Sperling et al. (2022) [27,28]
ABT-199	Bcl2i	A senolytic agent used in combined therapies that targets senescent cells for apoptosis through inhibition of an antiapoptotic factor, bcl2, which functions to sequester cytochrome c from the cytoplasm.	Liu et al. (2020) [10]
G3139		An antisense 18 mer phosphorothioate oligonucleotide that binds to bcl-2 mRNA and downregulates bcl-2, thus stimulating apoptosis.	Shah et al. (2009) [29]
YM155	Survivin inhibitor	YM-155 binds to interleukin enhancer binding factor 3 (ILF3) and disrupts transcriptional complexes at the survivin promoter to suppress transcription of the protein.	Dresang et al. (2013) [30]
BETd-246	BET degrader	Inhibits bromodomain proteins such as Brd2, Brd3, and Brd4, which drive transcription of oncogenes and promote proliferation of malignant tumor cells.	Choi et al. (2019) [31]
JQ1		A BET degrader that epigenetically silences genes via competitive binding with BET proteins and the displacement of these enzymes from acetylated lysine within the chromatin.	Shao et al. (2014) [32]
Copanlisib	PI3Ki	Inhibits an intracellular tyrosine kinase PI3K that is responsible for promoting cell proliferation, survival gene expression, differentiation, metabolism, and more.	Fang et al. (2020) [33]
Idelalisib		Specific inhibitor of the p110δ subunit of PI3K.	Chteinberg et al. (2018) [34]
BYL719		Specific inhibitor of the p110α subunit of PI3K.	Chteinberg et al. (2018) [34]
MLN0128	mTORi	mTOR functions within the PI3K pathway; the inhibition of mTOR reduces cell growth, proliferation, and survival.	Kannan et al. (2016) [35]
Olaparib	PARP1i	PARP1 and PARP2 inhibitor that prevents overactivation of the DNA damage repair pathway by tumor cells, thus reducing tumor cell survival.	Ferrarotto et al. (2018) [36]
3C2-1D12	Anti-ABCB5 mAb	Directly binds ABCB5, a multidrug resistance (MDR) mediator in various solid tumors.	Kleffel et al. (2016) [37]
Palbociclib	CDK4/6 inhibitor	CD4/6i that prevents progression through the cell cycle and increases programmed death ligand 1 (PD-L1) protein levels.	Lee et al. (2024) [38]
AK-01/LY3295668	Aurora Kinase Inhibitor (AURKi)	AURK inhibitors block the serine/threonine kinase activity of AURKs and subsequently prevent downstream activation of cell survival genes.	Das et al. (2021) [39]
CHIR99021	GSK3i	GSK3 functions in the ST-antigen regulation pathway and helps promote proliferation in MCPyV-positive MCC lines. CHIR99021 inhibits GSK3 function and subsequently causes the downregulation of ST-antigen expression.	Houben et al. (2022) [40]
Selinexor (KPT-330)	XP01i	Inhibits a specific exportin 1 transporter that LTA and STA mRNAs require to transport to the cytoplasm for translation.	Gupta et al. (2021) [41]
Trametinib	MEKi	Inhibitor of MEK1 and MEK2, both upregulators of growth gene expression and cellular proliferation.	Liu et al. (2016) [42]
NT-17	IL-7 mAb	Analog of IL-7 to increase the number of T-cells and enhance T-cell response to oncogenesis.	Wang et al. (2022) [43]
Ruxolitinib	JAKi	JAK is a non-receptor tyrosine kinase that promotes the proliferation of malignant cells and has been found to be upregulated in MCPyV-negative MCC. The inhibition of JAKi prevents constitutive activation of the JAK-STAT pathway.	Iwasaki et al. (2022) [44]

**Table 2:** Summary of in vitro and in vivo treatments of MCC cell lines with various targeted treatments.

Targeted Therapy	Type	Summary of Outcomes	References
Domatinostat	Epigenetic modifier	Domatinostat has been shown to upregulate antigen presentation on MCC cells in vitro and can be used to promote immunotherapeutic response.	Song et al. (2021) [23]
LSD1i		In vivo treatment of PeTa MCC cells with GSK-LSD1 for 6 days revealed markedly reduced tumor volume when compared with the vehicle. Annexin V and TUNEL staining revealed a ~2-fold increase in apoptosis for MCC cells treated with GSK-LSD1 as compared with the DMSO control group.	Leiendecker et al. (2020) [88]
Panobinostat		Panobinostat is an HDACi that led to an improved HLA class I expression and greater CD8+ T-cell infiltration in MCC tumor cells, though it did not seem to have a major clinical impact on two patients that had failed PD-1/PDL-1 blockade.	Ugurel et al. (2019) [25]
Nutlin-3	MDM2 inhibitor	The combination of MDM2 and MDM4 inhibition in the application of in vitro and in vivo mouse treatments of virus-positive cell lines resulted in significantly elevated p53 levels as well as more pronounced cell death than the vehicle following 96 h of treatment with the Lenalidomide-and-Nutlin-3 combination.	Park et al. (2019) [27]
Lenalidomide	MDM4 inhibitor		
ABT-199	Bcl2i	Five out of the eight MCPyV cell lines tested showed markedly reduced viability of MCC cells when treated with the synergistic combination of glaucarubin and ABT-199.	Liu et al. (2020) [10]
A1331852 WEHI-539	Bcl-xLi	The inhibition of B-cell lymphoma extra-large (Bcl-xL), which is encoded by Bcl2L1, results in antitumor effects in MCC cell lines, including a synergistic effect when poly (ADP-ribose) polymerase 1 (PARP) inhibitors are added.	Fan et al. (2023) [95]
Copanlisib	PI3Ki	Treatment with copanlisib showed a reduction in tumor size in in vivo mouse models as well as apoptosis in in vitro studies on MCC cells. In vitro treatment of MCC cells with BYL719 (alpelalisib) and idelalisib reduced cell viability after 120 h of treatment. BYL719 displayed more potent activity against MCC cells than idelalisib.	Fang et al. (2020) [33] Chteinberg et al. (2018) [34]
BYL719			
BETd-246	BET degrader	In vitro and in vivo applications of BET degraders in the treatment of 16 MCC cell lines revealed a reduced tumor volume of cells when treated with BETd-246 for 16 days.	Choi et al. (2019) [31]
JQ1		Treatment of MCC cell lines with JQ1 in vivo revealed decreased c-Myc expression and increased p21, p27, and p57, suggesting its role in inducing G1 arrest in MCC cells.	Shao et al. (2014) [32]
YM-155	Survivin (BIRC5i)	YM155 showed efficacy at low concentrations at inducing necroptotic cell death, but it acted more as a cytostatic agent in vivo, temporarily halting tumor growth without completely eliminating MCC tumor cells.	Donepudi et al. (2012) [98] Arora et al. (2012) [97]
Olaparib	PARP1i	In vitro investigations of Olaparib in the treatment of MCC cell lines revealed higher sensitivity to cell death in cell lines with increased PARP1 expression. While BRCA1/2 pathogenic variants are rare in MCC, PARPi may be a useful treatment in MCC that does show BRCA1/2 mutations.	Ferrarotto et al. (2018) [36] Gaubert et al. (2023) [106]
3C2-1D12	Anti-ABCB5 mAb	ABCB5 blockage in vivo reversed MCC drug resistance to carboplatin and etoposide, attenuated xenograft growth, and increased caspase 3 expression in mice injected with MKL-1 tumor cells.	Kleffel et al. (2016) [37]
Adcitmer®	Anti-CD56 mAb	Treatment of MCC xenograft mouse models with three injectable doses of Adcitmer ® led to statistically significant and sustained tumor volume reduction for ~30 days. Minimal to no toxicity was observed as a consequence of the treatment.	Esnault et al. (2022) [22]
Palbociclib	CDK4/6i	When MCC cells were co-treated with Palbociclib combined with TC-S7009, an HIF2α inhibitor, increased PD-L1 expression was impeded, resulting in increased reactive oxygen species and cell death via ferroptosis.	Lee et al. (2024) [38]
AK-01 (LY3295668)	AURK inhibitor	A total of 6 MCC cell lines and 2 xenograft mouse models were treated with AK-01. CCK-8 assays revealed decreased proliferation of MCC cells in vitro by ~50% at doses >100 nM. Xenograft mouse models showed an increased proportion of cells that entered G2 when treated with AK-01, suggesting G2-M transition failure in these cell lines.	Das et al. (2021) [39]
CHIR99021	GSK3 inhibitor	In vitro and in vivo inhibition of GSK3 in MLK-1 (MCPyV-positive MCC) xenograft tumor mice led to the suppression of MCC tumor size, although complete cessation of growth was not observed. Importantly, treatment of mice with the GSK3 inhibitor did not precipitate any visual signs of toxicity, indicating its potential to be translated into clinical trials and potentially combined with another ST-antigen inhibitor to synergistically prevent the proliferation of MCC cells.	Houben et al. (2022) [40]
Selinexor	XP01i	Displayed antiviral activity in MCC MCPyV-positive cell lines by downregulating expression of the virus.	Gupta et al. (2021) [41]
Trametinib	MEKi	Found that EGF and FGF are required to promote MCPyV infection of human dermal fibroblasts, inhibition of the MAPK pathway, and subsequent growth factor activation. Trametinib also prevents MCPyV infection.	Liu et al. (2016) [42]
Ruxolitinib	JAKi	In vitro treatment of both MCPyV-positive and -negative MCC cell lines with (50–500 uM) of ruxolitinib yielded significant inhibition of cell growth and prevented ERK phosphorylation, as confirmed through WST8 cell viability assays and Western blots.	Iwasaki et al. (2022) [44]
Tazemetostat	EZH2i	Mice with MCC xenograft tumors were treated with the EZH2i Tazemetostat for 50 days, and the results revealed delayed tumor growth upon treatment with the agent, without complete inhibition of tumor growth over time.	Gartin et al. (2022) [26]

**Table 3:** Potential targets for treatment of MCC based on mechanistic studies.

Proposed Target	Summary of Outcomes	References
p-ALK	p-ALK was identified in ~47.8% of MCC cell samples tested with IHC and could be a potential target for treatment, as ALK has been shown to contribute to the pathogenesis of various tumors such as MCC. Not only can ALK be targeted therapeutically, but it can also act as a prognostic marker, as p-ALK correlates with MCPyV positivity, younger patients, and non-metastatic lesions.	Jaatinen et al. (2021) [107] Cao and Namburidi (2017) [108]
PLK	Polo-like kinase 1 (PLK1) was found to be expressed in a significant proportion of patients, suggesting its prominent role in the disease’s pathogenesis and potential as a therapeutic target. The potential of PLK1 inhibitors, such as BI2536, offers a promising therapeutic avenue.	Kadletz et al. (2016) [109]
BRD4	BRD4 has been shown to interact with the truncated MCV LT antigen, aiding viral replication. BRD4 could act as a potential target to reduce MCV viral load.	Arora et al. (2019) [110]
ADAM 10, ADAM 17	A previous investigation performed by Nwogu et al. (2018) found that MCPyV ST antigen expression promotes upregulation of the ADAM 10 and 17 proteins, which are disintegrins and metalloproteinases partly responsible for the detachment and metastasis of MCC tumor cells. Targeting of ADAM 10 and 17 could reduce the metastatic potential of MCC tumor cells.	Nwogu et al. (2018) [111]
KOC	KOC, also known as L523S or IMP-3, is an insulin-like growth factor II messenger RNA-binding protein involved in promoting tumor cell proliferation. Upon analyzing 20 MCC specimens for KOC expression using immunohistochemistry, a significant majority (90%) of MCCs expressed KOC, with many showing moderate to strong immunostaining intensity. The degree of KOC expression might be indicative of the tumor’s potential to metastasize, thereby helping in the stratification of patients for more aggressive treatment and closer monitoring.	Pryor et al. (2009) [112] Lien et al. (2010) [113]
CCL17/TARC, CCR4	CCL17/TARC and CCR4 upregulated in MCC MCPyV-positive cell lines, which contributed to the constitutive activation of the MAPK and NF-KB pathway and the stimulation of MCPyV promoter activity. Taken together, CCL17/TARC and CCR4 act as a potential target for therapy.	Rasheed et al. (2018) [114]
trKA	A case series involving 55 patients was performed to understand whether anti-tropomyosin receptor kinase A was upregulated in MCC and revealed that trKA was present in all cell lines used in the study.	Wehkamp et al. (2017) [115]
c-Jun	c-Jun phosphorylation promotes MCPyV ST antigen activity, which helps upregulate proliferation of MCPyV MCC-positive cells. Inhibiting c-Jun could potentially reduce viral load and prevent oncogenic proliferation.	Wu et al. (2016) [116]
Mcl-1	The high expression of Mcl-1, an antiapoptotic protein, and Bmi-1, a transcriptional repressor, in MCC samples suggests they could be targets for antisense oligonucleotide therapies. This could be a novel approach in treating MCC by inhibiting genes essential for tumor growth and survival.	Brunner et al. (2008) [54]
RNAi	RNA interference (RNAi) may serve as a targeted therapeutic strategy against MCPyV-positive MCC cases. RNAi, a gene-silencing mechanism, uses small double-stranded RNA to target and degrade specific mRNA molecules. This process can inhibit the expression of essential viral proteins like the T antigen in MCV, crucial for the virus’s role in cancer development.	Hoque et al. (2012) [117]
CADM1	CADM1 expression is typically lower in MCPyV-positive cases compared to MCPyV-negative cases. Higher CADM1 expression correlates with poorer outcomes in MCC, indicating its potential role as an oncoprotein in MCPyV-negative MCCs. This finding suggests the diverse functions of CADM1 in MCC and its possible significance in developing targeted therapies.	Iwasaki et al. (2016) [118]
TERT	Telomerase reverse transcriptase (TERT) has been identified as a crucial factor in the progression of MCC. Mutations in the TERT promoter are more frequent in sun-exposed areas and MCPyV-negative tumors. These mutations and increased TERT mRNA expression correlate with poorer survival outcomes. This underscores the potential of TERT as a therapeutic target in MCC treatment.	Miles and Saini (2015) [119]
RB1	RB1, often inactivated in MCC, especially in cases without MCPyV, can be a potential target. Its inactivation is pivotal in MCC pathogenesis, suggesting therapies that could counteract this effect.	Erstad et al. (2014) [120]
TP53	TP53, although rarely mutated in MCC, has an altered expression in some cases, and its role in the cell cycle and apoptosis makes it a potential target for therapies that could restore or mimic its tumor-suppressive functions.	Erstad et al. (2014) [120]
Stathmin	Stathmin, a microtubule-associated protein, is overexpressed in various cancers, including MCC, and is linked to poor prognosis and high metastatic potential. Small T antigens (ST) promote microtubule destabilization and lead to increased levels of unphosphorylated stathmin, thereby enhancing microtubule destabilization and cell motility. Targeting stathmin through siRNAs is suggested as a potential strategy to inhibit the metastatic capability of MCC.	Knight et al. (2015) [121]
EMT pathway	Karpinski et al. (2023) propose focusing on the epithelial mesenchymal transition (EMT) pathway, a biological process in which epithelial cells develop factors leading to cancer progression, may be a potential target particularly in MCPyV-negative MCCs, as these MCCs demonstrated higher expression of EMT-associated genes.	Karpinski et al. (2023) [122]
DLL3	DLL3 is a protein involved in neurogenesis during embryonic development and has been found to be expressed in 91% of MCPyV-positive MCC cases. Treatment of small-cell lung cancer patients with Rovalpituzumab tesirine, a DLL3 inhibitor, led to antitumor activity and indicates its possibility of being applied in MCC therapies as well.	Esnault et al. (2022) [95]
BRCA1/2	Although there is a low frequency (3%) of BRCA1/2 pathogenic variants in MCC, poly-(ADP-Ribose)-polymerase inhibitors could be effective for patients with BRCA-mutated MCC, though this requires further investigation.	Gaubert et al. (2023) [106]
ECM	In a study of ECM composition of 11 MCC tumors, second-harmonic-generation (SHG) microscopy revealed profound changes in the collagen structure of these tumors. Specifically, SHG revealed thinner, more homogenous collagen fibers in MCC samples, and this variation indicated the possibility that collagen remodeling may play a role in the aggressiveness of this tumor type.	Laurito et al. (2021) [123]
GPC3	Glypican-3 (GPC3) is a tumor antigen expressed in MCC, particularly in MCPyV-negative cases that are more aggressive and is another potential drug target.	Muralidharan et al. (2022) [124]
pmCiC	Plasma membrane citrate transporter (pmCiC), which is involved in cancer cell proliferation and spread, has been shown to be upregulated in MCC cell lines and may serve as another drug target.	Drexler et al. (2022) [125]
MUC-1	The inhibition of MUC-1, a protein that promotes MCC cell survival, is another therapeutic approach demonstrated in MCC cell line studies.	Morimoto et al. (2022) [126]
PP4R1	PP4R1 has been found to form a complex with other regulators, such as the MCC MCPyV small T antigen, PP4C, and the NEMO adaptor protein, to deactivate the NF-KB pathway. Interactions between PP4R1 and NEMO are specific to MCPyV and thus can act as a target for treatment.	Abdul-Sada et al. (2017) [127]
PDGFRA	Platelet-derived growth factor receptor alpha (PDGFRA) was found to be expressed in ~87% of MCC cases and is a member of the type III receptor tyrosine kinase family, just like c-KIT [45]. This suggests its potential to be targeted with TKIs similar to imatinib.	Kartha et al. (2008) [45] Swick et al. (2013) [51]

## Data Availability

The contributions presented in this review have been summarized in the Section 2. Further inquiries may be directed to the corresponding author.

## Supplementary Materials

The following supporting information can be downloaded at https://www.mdpi.com/article/10.3390/ijms252011055/s1.
