# Supplementary material for: Advancing Treatment Options for Merkel Cell Carcinoma: A Review of Tumor-Targeted Therapies
Source: Int J Mol Sci. 2024 Oct 15;25(20):11055. doi: 10.3390/ijms252011055 (PMC11507330; doi:10.3390/ijms252011055)
Supplement: Supplementary file 1 [file ijms-25-11055-s001.zip › ijms-3186999-supplementary.pdf]

Supplementary Table S1. Clinical Trials and associated studies surrounding use of targeted therapies to treat Merkel cell carcinoma patients.

Therapy	Type	Summary of Outcomes	Study
Imatinib	Receptor TK inhibitor	A 92-year-old woman with MCC experienced complete tumor resolution after two months of treatment with imatinib.	Loader et al. (2013) [48]
		A phase II trial assessing the efficacy of imatinib mesylate in 23 individuals with metastatic or unresectable MCC found minimal therapeutic impact. Only one partial response among 23 participants was observed.	Samlowski et al. (2010) [14]
		A 77- year-old man with MCC was treated with 400 mg of imatinib twice daily for 6 months. The patient experienced no recurrence of the disease for 16 months following the cessation of imatinib treatment; however, his treatment was complicated by grade 3 ageusia and asthenia, which led to a reduction in dosage of the drug to 400 mg daily.	Peuvrel et al. (2011) [56]
Pazopanib		A 69-year-old woman with metastatic MCC treated with pazopanib achieved a complete clinical response in her scalp lesion and a partial response in pulmonary metastases.	Davids et al. (2009) [59]
		A phase II trial studying pazopanib in patients with advanced MCC who had been either pre-treated with chemotherapy or naive was halted prematurely once the primary objective was met and due to slow accrual. Of the 16 patients that participated, 3 exhibited partial responses meanwhile 6 experienced stable disease progression.	Nathan et al. (2016) [61]
		5 patients with MCC treated with pazopanib experienced initial improvements but ultimately faced disease progression.	Tarabadkar et al. (2018) [60]
		Out of 11 patients with advanced, pre-treated MCC who were administered Pazopanib, 4 patients (36%) experienced 7-13.6 mo. of stable disease progression.	Knepper et al. (2021) [62]
Cabozantinib		In a phase II trial assessing the efficacy and safety of cabozantinib in advanced MCC, only one patient out of 8 achieved stable disease for 8 months. The study was terminated due to toxicity.	Rabinowits et al. (2018) [63]
		A clinical study consisting of 5 patients with MCC found that treatment with pazopanib led to 3 mo. of disease-free survival for 1 patient and stabilization of MCC from 5 months to 3.5 years in 4 participants.	Tarabadkar et al. (2018) [60]

		An 86-year-old man with metastatic medullary thyroid cancer was diagnosed with stage III MCC, another primary tumor, and was treated with 100 mg then 60 mg of cabozantinib, which resulted in partial response on imaging. Treatment was interrupted due to toxicity concerns.	Zago et al. (2023) [16]
Apatinib		An 86-year-old man with metastatic MCC was treated with endostar and apatinib and experienced progression-free survival for 6.5 months with 13.0 months overall survival.	Jiang et al. (2019) [64]
Lenvatinib		Ongoing phase II clinical trial studying neoadjuvant treatment with Lenvatinib + pembrolizumab in stage II-IV MCC patients.	NCT04869137
Axitinib		Active, not recruiting clinical trial studying the effects of axitinib with sansanlimab (anti-PD1 antibody), and PF-07265807 which targets AXL and MERTK thereby inhibiting tumor-associated macrophage kinases.	NCT04458259
Octreotide	Somatostatin analog (SSA)	Of 19 patients with metastatic MCC receiving SSA treatment, 7 responded positively and 3 responded with prolonged stabilization of the disease over several months.<br><br>A 73-year-old man with recurrent MCC was treated with octreotide and avelumab. Further growth or progression of the tumor was not observed at the end of a 3-year treatment regimen.	Akaike et al. (2021) [67]<br><br>Guida et al. (2020) [65]
Lanreotide		A patient with metastatic MCC showed a complete response to octreotide treatment lasting ten months.<br><br>In a study on the efficacy of SSA treatments in patients with MCC, an 87-year-old female achieved 17 mo. of clinical remission of MCC when treated with Lanreotide. The treatment was tolerated well, and no side effects were observed.	Cirillo et al. (1997) [69]<br><br>Fakiha et al. (2010) [71] Brummer et al. (2016) [68]
90Y-DOTATOC		An 81-year-old female patient with recurrent MCC was treated with 90Y-DOTATOC and experienced complete clinical remission within a week after the initial dose, and periods of remission were induced upon subsequent treatments as well.	Meier et al. (2004) [66]
PPRT		A phase II trial is being performed to assess efficacy of Peptide receptor radionuclide therapy (PPRT) in patients with different neuroendocrine tumors. This treatment mechanism includes SSAs 177Lu-DOTATATE and ^{68}Ga-DOTA-Tyr3-octreotide.<br><br>A clinical trial examining combination therapy with avelumab and 177-Lu-DOTATATE is in progress.	Cassler et al. (2016) [75]<br><br>NCT04261855

Navtemadlin	MDM2i	A phase 1/b clinical trial in 31 patients with p53 wild type MCC showed a 25% confirmed objective response rate (ORR) and a 63% disease control rate among those receiving treatment.	NCT03787602
IMGN901	Anti-CD56 Ab	Patients were administered IV IMGN901 every 3 days for 21 days. The maximum dose tolerated was 75 mg/m2. While overall a tolerable therapy, adverse side effects included fatigue, neuropathy, headache, chest pain.	Shah et al. (2016) [20]
NT-17	IL-7 mAb	Ongoing clinical trial that is using NT-17 in combination with pembrolizumab (PDL-1 inhibitor) on cancer patients including those with stage III-IV MCC.	NCT03901573
G3139	Bcl2i	A Phase II trial found that G3139 was well tolerated in MCC patients but showed no objective response in 11/12 of the patients involved in the study.	Shah et al. (2009) [29]
Idelalisib	PI3Ki	An 86-year-old woman with stage IIIB MCC resistant to chemotherapy achieved complete clinical remission with idelalisib administered at 150 mg twice daily.	Shiver et al. (2015) [29]
MLN0128	mTORi	A phase I/II clinical trial was carried out to assess the toxicity of MLN0128 in 9 patients with MCC. 1 patient withdrew due to toxicity of the drug treatment.	Femia et al. (2018) [84], NCT02514824
